# A Delayed Onset of Lateral Medullary Syndrome: A Case Report

**DOI:** 10.7759/cureus.78956

**Published:** 2025-02-13

**Authors:** Lexi Garber, LaTeya Foxx

**Affiliations:** 1 Department of Osteopathic Medicine, Lake Erie College of Osteopathic Medicine, Bradenton, USA; 2 Department of Research, Lake Erie College of Osteopathic Medicine, Bradenton, USA; 3 Department of Neurology, Baptist Neurology Group, Jacksonville, USA

**Keywords:** ischemic stroke, lateral medulla, lateral medullary syndrome (wallenberg syndrome), stroke evolution, stroke treatment

## Abstract

Lateral medullary syndrome (LMS), also known as Wallenberg syndrome, is a rare stroke that commonly affects the posterior inferior cerebellar artery and, less commonly, the vertebral arteries. LMS presents with a common constellation of posterior circulation stroke symptoms. In this case, we are discussing a 72-year-old male patient who has been diagnosed with left LMS. While the patient’s presentation at the time of the suspected diagnosis included classic symptoms of Wallenberg syndrome, his initial visit to the emergency room five days before did not, as he only presented with vertigo symptoms: dizziness, a spinning sensation, and balance issues. Therefore, the delayed presentation of the patient's symptoms will be discussed in this case report. It took five days for typical lateral medullary symptoms to occur, which is unusual for ischemic strokes, especially LMS. While it is unusual for symptoms to occur gradually, it raises an important concept of stroke evolution, which describes how strokes can progress over a period of time either due to worsening ischemia or extension of the infarct. Stroke evolution is clinically significant because it emphasizes that stroke symptoms are not always forthcoming and direct, as highlighted in our patient's case. Even with initial negative diagnostic studies, clinicians should not completely rule out LMS or other types of ischemic strokes given the idea of stroke progression. Our patient was diagnosed with LMS initially on the basis of physical examination alone and later confirmed by a repeat diffusion-weighted MRI.

## Introduction

Lateral medullary syndrome (LMS) occurs due to thrombosis or embolization of the posterior inferior cerebellar artery, leading to infarction of the lateral medulla [1]. LMS commonly presents with ipsilateral ataxia, nystagmus, diplopia, dysphagia, dysarthria, vertigo, hoarseness, singultus, ipsilateral Horner’s syndrome, and mixed sensory deficits [2]. Due to the involvement of the lateral spinothalamic tract, there is a loss of pain and temperature on the ipsilateral side of the face and the contralateral side of the body [1]. There can be ipsilateral Horner’s syndrome due to the involvement of the descending sympathetic tract. Due to the involvement of the nucleus ambiguus, there can be dysphagia, dysarthria, and hoarseness. Singultus, which is commonly overlooked in LMS, is believed to be caused by the involvement of the nucleus ambiguus or surrounding areas [3]. Also, with vestibular nuclei involvement, patients can experience nystagmus, nausea, vomiting, and vertiginous symptoms. It is also common to have cerebellar ataxia due to the involvement of the inferior cerebellar peduncle [1]. Some of the risk factors associated with LMS are atherosclerosis, hypertension, and cardiac diseases, such as atrial fibrillation [3]. Another study also found that these types of infarcts are commonly seen in those who consume alcohol [4]. The onset of LMS is usually sudden in patients. Using physical exam and patient history, the clinician can suspect LMS, but it should be confirmed with diagnostic testing, such as CT and MRI brain with diffusion-weighted magnetic resonance imaging, the best for detecting early infarcts. Computed tomography angiography (CTA) and magnetic resonance angiography are more advanced forms of arterial imaging used to identify which vessels are involved [3]. Although the onset of LMS is usually sudden, it took five days for our patient to present with typical LMS symptoms. Therefore, the concept of stroke progression will be discussed in the case report and how it is relevant to our patient's delayed diagnosis.

## Case presentation

A 72-year-old male patient with a history of hypertension, dyslipidemia, and chronic pain presented to the emergency department with sudden onset feelings of vertigo, spinning sensation, dizziness, and balance issues, feeling like he was “swaying toward the left side.” He also admitted to having feelings of shakiness, sweatiness, and nausea. His initial blood pressure was elevated at 196/100, heart rate was 58 bpm, and respirations were 16/minute. The patient denied symptoms like this in the past. He reported being compliant with his antihypertensive medications: clonidine 0.3 mg three times daily, amlodipine 10 mg once daily, and losartan/hydrochlorothiazide 100/25 mg once daily. EKG was done and revealed sinus bradycardia. CT without contrast of the head was ordered, and no signs of acute stroke were found, although there was an age-indeterminate left paramedian pontine infarct. CTA of the head and neck revealed bilateral carotid mild atherosclerotic plaque and carotid calcification with approximately 50% left internal carotid artery narrowing with no significant internal carotid artery stenosis. MRI of the brain revealed no acute intracranial abnormality and no acute infarct. Aspirin was given in the emergency department, and neurology was consulted due to possible stroke, along with nephrology for uncontrolled hypertension and cardiology to perform a transthoracic echocardiogram.

Neurology originally suspected a possible transient ischemic attack (TIA), as the patient’s initial symptoms of dizziness, sweatiness, and veering to the left had improved without any obvious neurologic deficits, with the exception that gait was not assessed due to fall precautions awaiting gait assessment by physical therapy. The initial MRI of the brain did not reveal any acute infarcts. The patient was continuously being monitored in the hospital to manage uncontrolled hypertension and new-onset bradycardia. An echocardiogram revealed an ejection fraction of 55%-60% with a negative bubble study, although calcification of the aortic valve was noted, likely due to atherosclerosis. Telemetry showed sinus bradycardia with a 2:1 atrioventricular conduction block with intermittent Wenckebach. EKG showed sinus bradycardia. His labs demonstrated hypercalcemia, mild hyponatremia, and a serum creatinine of 0.9. The patient denied taking nonsteroidal anti-inflammatory drugs, tobacco use, or alcohol use. Nephrology ordered a renal ultrasound, which showed possible renal artery stenosis and atherosclerosis of the left renal artery and some right renal cysts. The patient was also noted to have dyslipidemia, although low-density lipoprotein was 88 and high-density lipoprotein was 38. The patient had been compliant with his statin medication and fenofibrate.

During his hospitalization stay, the patient developed hypotension, elevated blood urea nitrogen, creatinine, and lactic acidosis. He was diagnosed with acute kidney injury due to sepsis and was started on vancomycin and IV fluids. IV heparin was also started due to increasing troponins. Also, during the patient’s hospital stay, occurring around day 5, he developed a sudden onset headache and diplopia, with new onset dysphagia, hoarseness, and weakness of his left side, feeling like he was veering toward the left side. The patient also began to have intractable singultus. The patient also developed dysarthria, and the patient’s wife said she could not understand what he was saying. Due to this new constellation of symptoms, neurology was reconsulted at this time.

On a neurologic exam, the patient was found to have bilateral horizontal and vertical nystagmus. He was also noted to have bilateral diplopia, decreased muscle strength in the left trapezius, abnormal heel-to-shin testing, and abnormal finger-to-nose testing on the left. The patient was also visibly having intractable singultus and dysphagia but did not seem to have any issues with speech. Muscle tone was normal in all four limbs. Plantar reflexes were normal bilaterally. Deep tendon reflexes were 2+ with no evidence of clonus. Pupils were round, equal, and reactive to light bilaterally. Hearing was normal bilaterally. The sensory exam did demonstrate contralateral vibration and temperature loss in the trunk. There were no tongue fasciculations and no deviation of the uvula.

Due to the alarming new symptoms and the patient’s neurological examination, a stat CT and MRI brain were ordered. Rectal aspirin was administered due to the patient’s dysphagia. He was not a candidate for thrombolytic therapy (tenecteplase or alteplase) due to the unclear onset of symptoms. The patient continued to experience some subjective symptoms throughout his admission and had not achieved a complete resolution of his initial complaints.

**Figure 1:**
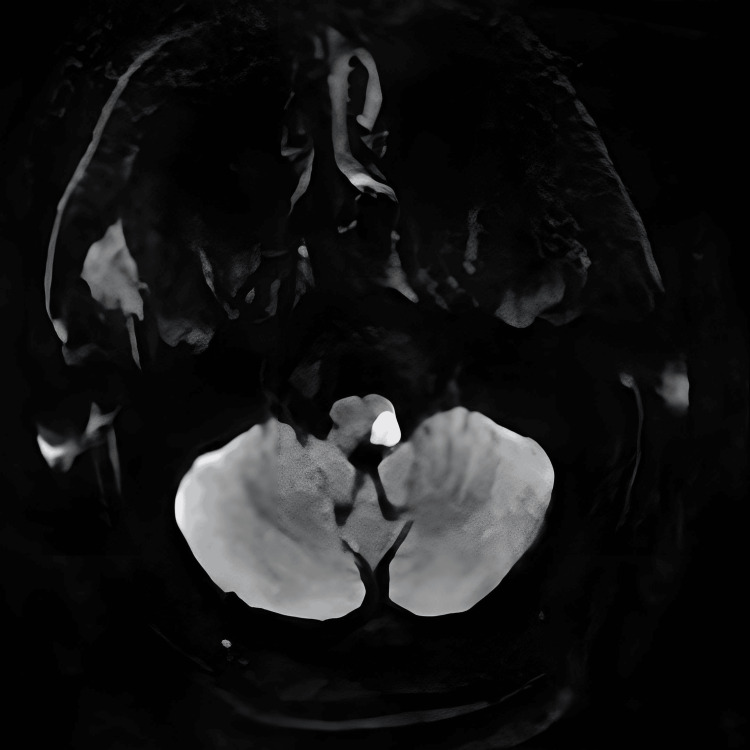
Diffusion-weighted axial MRI showing restricted diffusion involving the left medullary region, consistent with a subacute left lateral medullary infarct

The CT results showed no acute intracranial abnormalities and no changes in the chronic lacunar ischemic infarct in the midbrain that the patient had previously experienced. As seen in Figure 1, a diffuse weighted axial MRI was done and showed restricted diffusion involving the left medullary region, consistent with a subacute left lateral medullary infarct.

One day after aspirin and clopidogrel administration, some of the patient’s symptoms slightly improved. Nystagmus and double vision were both still present but improving. Dysarthria resolved, and muscle weakness improved as well. The patient still felt ataxic. Severe dysphagia and singultus persisted. Due to severe dysphagia, a Dobhoff tube was placed in the right nare.

## Discussion

Stroke evolution and LMS

LMS is an uncommon stroke to diagnose in clinic [5]. Wallenberg syndrome only accounts for around 2% of hospital admissions for acute strokes [6]. Although the constellation of symptoms that patients experience are common, such as dysphagia, nystagmus, and vertigo, the timing of our patient’s symptoms is quite unique. As discussed in the case presentation, our patient did not initially present with classic LMS symptoms upon arrival in the emergency department. Our patient’s main symptoms were vague and vestibular-related, including balance issues and dizziness, along with the feeling of the room spinning. After the improvement of these symptoms, it took around five days from the time of admission to the onset of left-sided weakness, singultus, and dysphagia to develop. This gradual timing of around five days for classic symptoms to develop is uncommon for this type of stroke. Studies reveal that the onset of LMS is sudden in 75% of cases and gradual in only 25% of patients [3]. This idea highlights the uniqueness of the gradual development of symptoms in our patients. It also highlights that even with vague vestibular systems initially and negative initial diagnostic MRI/CT, this type of stroke should still be considered in symptomatic patients. Signs should be carefully monitored for possible gradual or stuttering progression into more indicative symptoms, as in our patient’s case.

There is another case study in which there was a delay in the diagnosis of LMS. It took around two days for symptoms to gradually appear in that case [7]. It was also found that posterior circulation strokes and lateral medullary strokes, in general, are frequently missed due to vague symptoms and negative initial imaging findings [7]. In fact, it was found that between 28% and 59% of these strokes are misdiagnosed and are two to three times more likely to be missed compared with anterior circulation strokes [8]. The study also highlights that once other causes have been ruled out for dysphagia, neurogenic origins should be revisited, as many patients with this type of stroke might only present with dysphagia as their only symptom [7]. There are many different variations of LMS, and symptoms might be vague and take days to progress.

Once fully progressed, our patient developed the classic LMS symptoms, such as ataxia, nystagmus, dysphagia, dysarthria, hoarseness, singultus, diplopia, and decreased muscle strength. Given our patient's physical examination and symptoms at this time, a third MRI brain was ordered during his hospital stay, and it confirmed his diagnosis of LMS, as discussed in Figure 1. MRI with diffusion-weighted imaging is the gold standard for confirming Wallenberg syndrome [9]. CT was also done to rule out any hemorrhagic lesions and to visualize structures in the posterior fossa, but it is often normal in those with LMS. Discussing the progression of our patient’s symptoms is crucial, as it highlights the idea of stroke evolution. Stroke evolution, or progressing stroke, was first discussed in the 1960s and highlights the idea that a patient with a neurologic deficit can gradually or in a stuttering fashion progress over a period of hours while the clinician sees the patient [10]. Stroke progression is often believed to be due to the worsening of focal ischemia, extension of the infarct, or even slow onset of hemorrhage. Atherosclerotic thrombosis of large arteries is known to play a role in progressive strokes, along with cardiac embolism, distal field infarction, and lacunes. Whatever the etiology, on physical exam, there will be a noticeable increased severity of neurologic deficits. It was also found that the timing of symptoms can present 36-48 hours after the actual infarction ensues, with late exacerbations up to seven days after the onset of initial symptoms. It took around five days for the increased severity of neurologic deficits to occur in our patient’s case. While infarcts in the carotid region might present differently than in the vertebrobasilar regions, both locations can produce progressive strokes. A stuttering TIA can also be considered based on the patient’s history, but the presence of our patient’s symptoms persisting for longer than 24 hours makes evolved ischemic infarct more probable.

This concept of stroke evolution is clinically significant because it emphasizes that stroke symptoms can be vague at initial presentation but later progress to a more typical presentation over a period of hours to days. With our patient's symptoms of dizziness, vestibular-related issues, and negative diagnostic studies initially, LMS was not a compelling differential diagnosis at the time. Likely due to worsening ischemia or extension of the infarct, typical LMS symptoms developed, such as singultus, dysphagia, dysarthria, and nystagmus. Even with initial negative diagnostic studies, clinicians should not completely rule out LMS or other types of ischemic strokes due to the idea of stroke progression. As highlighted in our patient's case, repeat imaging and physical examination should be considered even after several days.

Risk factors and diagnostic challenges

Hypertension is a risk factor for LMS, although our patient was compliant with his medications [3]. His uncontrolled hypertension could be from his renal artery stenosis, as three medications were not able to properly control the patient’s blood pressure. His past medical history of hypertension is still a risk factor for his diagnosis. The patient’s hypertension/hypotension and bradycardia/tachycardia throughout his hospital stay may also be due to autonomic dysfunction related to baroreceptors located in the medulla [11]. After completing further investigation of the patient, there was noted to be renal artery stenosis, carotid artery atherosclerotic plaques, and a calcified aortic valve. The patient has a degree of atherosclerosis throughout his cardiovascular system. Atherosclerosis is another major risk factor for developing this type of stroke and the progressive timing of symptoms [3]. Atherosclerotic plaques can cause ischemia by restricting blood flow to the arteries in our body. If these plaques build up in the posterior inferior cerebellar or vertebral arteries, it can cause blockage, creating ischemia and causing characteristic symptoms of LMS [3]. Interestingly, the patient had evidence of an age-indeterminate pontine infarct on CT, which was confirmed to be chronic on MRI brain. This suggests that the patient had a previous asymptomatic ischemic event, as he denied a history of cerebrovascular accident and was not on any anticoagulation medications at the time of admission.

Treatment considerations

Our patient was treated with dual antiplatelet therapy, including both aspirin and clopidogrel. In some cases, if the presentation is early enough, tissue plasminogen activator can be used [12]. As mentioned in the case presentation, some of the patient’s symptoms slightly improved, even after only one day. Nystagmus and double vision were both improving. Dysarthria resolved, and muscle weakness improved as well. Severe dysphagia and singultus persisted after one day of treatment. Although some patients restore swallowing function quickly, dysphagia from a lateral medullary stroke is often more severe and may not resolve for months or even years [13].

## Conclusions

LMS is an uncommon stroke to diagnose in clinical practice and can present in many variations. Although there are classic symptoms associated with this syndrome, they may not appear initially. Given that strokes can evolve over several days, it is reasonable to continue with a high degree of suspicion for ischemia, especially in patients without full symptom resolution, who also have significant stroke risk factors. Initial CT and MRI brain testing may not always identify early phases of acute CNS ischemia, which can occur in up to 50% of cases, as in our patient’s case. In fact, our patient had three MRIs before showing positive signs. This highlights that there are different variations of LMS and patients can have a delayed presentation. However, it is crucial to remain vigilant and keep stroke in the differential diagnosis even with initial negative diagnostic studies. It is essential to monitor these patients for signs of progression and promptly repeat physical examination and diagnostic imaging to contribute to a better prognosis. Early and accurate diagnosis can improve treatment and contribute toward better patient outcomes.
